# Neurocognitive mechanisms underlying action tool knowledge tasks: specificity of tool-tool compared to hand-tool compatibility tasks

**DOI:** 10.1038/s42003-025-07923-1

**Published:** 2025-04-03

**Authors:** Mathieu Lesourd, François Osiurak, Julie Martin, Sébastien Hague, Margolise Laroze, Gautier Clément, Elisabeth Medeiros de Bustos, Guillaume Fargeix, Eloi Magnin, Thierry Moulin

**Affiliations:** 1 Université Marie et Louis Pasteur, INSERM, UMR 1322 LINC, F-25000 Besançon, France; 2 Maison des Sciences de l’Homme et de l’Environnement (UAR 3124), Besançon, France; 3 Unité de Neurologie Vasculaire (UNV) et Hôpital de jour (HDJ), Service de Neurologie, CHRU de Besançon, Besançon, France; 4 Laboratoire d’Étude des Mécanismes Cognitifs (EA 3082), Université Lyon 2, Bron, France; 5 Institut Universitaire de France, Paris, France; 6 Centre Mémoire Ressources et Recherche (CMRR), service de Neurologie, CHRU Besançon, F-25000 Besançon, France

**Keywords:** Human behaviour, Cognitive neuroscience

## Abstract

Action tool knowledge can be assessed mainly with two kinds of tasks: tool-tool and hand-tool compatibility tasks. While these tasks are used to assess action tool knowledge, recent data showed striking dissociations between these tasks in brain-damaged patients. In this study, we explored the neuropsychological dissociations (Experiment 1; 60 brain-damaged patients) and the potential cognitive mechanisms engaged during these two tasks (Experiment 2; 52 healthy participants). Finally, we also reanalyzed fMRI data to investigate the neural bases engaged in tool-tool and hand-tool compatibility tasks (Experiment 3; 34 healthy participants). The three experiments provide convergent arguments by showing that both tasks share common core computations supported by a left-lateralized brain network, but hand-tool compatibility task engages regions outside of this brain network and is explained by visual imagery while tool-tool task is rather explained by motor imagery. Our results shed a new light on action tool knowledge tasks.

## Abstract (web summary)

This study explores the neurocognitive mechanisms of action tool knowledge tasks. Behavioral, cognitive, and neuroimaging data reveal shared processes but distinct reliance on motor vs. visual imagery for tool-tool and hand-tool compatibility tasks.

## Introduction

Studying the neurocognitive basis of tool use in healthy subjects and brain-damaged patients has been the subject of extensive research over the past decades. Most cognitive models assume that, in order to use a tool, people have to form a representation of the action to be performed with it (i.e., conceptual stage). These representations, also called manipulation knowledge, contain information about how to manipulate familiar tools, i.e., enabling us to grasp tools in a correct fashion and using them in a meaningful way^1^. Manipulation knowledge is assessed using two types of tasks interchangeably: tool-tool compatibility tasks and hand-tool compatibility tasks. However, this approach may be problematic because dissociations observed in left brain-damaged (LBD) patients suggest that these two tasks engage distinct neurocognitive processes. The aim of this work is to better understand these mechanisms.

Tool-tool compatibility tasks consist in asking participants to decide whether two tools are grasped (i.e., hand posture component^2^) or manipulated in the same way (i.e., kinematic component^3^). While participants are explicitly asked to focus on either hand posture or on kinematic in some studies^4,5^, other studies do not distinguish between these two components of action, considering manipulation as a whole^6–8^. Thus, it is likely that when participants are asked to judge whether two tools are manipulated in the same way, they preferentially focus on the kinematics component of the action rather than the hand posture component. In hand-tool compatibility tasks, also called recognition of gesture tasks^9,10^, participants have to decide whether a tool is correctly held in hand among several distractors^9–12^. In similar tasks, participants have to choose among several hand postures the one that is suitable for grasping a given tool^13,14^. Thus, in hand-tool compatibility tasks, subjects are asked to choose the correct hand that fits with a given tool in order to use it, therefore focusing on the hand posture component of action. Concerning neuroimaging studies, it has been reported almost systematically a coactivation of the left IPL and left pMTG in both tool-tool and hand-tool compatibility tasks^15^, indicating that both tasks are relying upon similar temporo-parietal brain networks^16–18^.

Although these two tasks are traditionally used interchangeably to assess the integrity of manipulation knowledge and are both engaging similar brain networks, there is some evidence that hand-tool and tool-tool compatibility tasks are supported by distinct neurocognitive processes, at least in part. For instance, neuropsychological data showed that in LBD patients, a striking dissociation in manipulation tasks with tool-tool compatibility tasks (difference control-patients: range = 15–48.1%) being more impaired than hand-tool compatibility tasks (difference control-patients range =  3.4–11.4%)^15^. Currently, the neuropsychological dissociations obtained at the group level, with lower tool-tool scores compared to hand-tool scores, may indicate that compatibility tool-tool task is more resources demanding compared to hand-tool compatibility task, or it may be more sensitive to a left-brain lesion.

The tool-tool compatibility task can be considered more cognitively demanding, as it requires the activation of kinematic components and the comparison of these activated components for each tool, while being maintained in working memory. Moreover, the absence of the hand in the tool-tool compatibility task may render mandatory the reenactment of the hand and planning the functional grasp of the hand’s position on the tool to mentally compare actions associated with the two tools^15,19–21^. In line with this hypothesis, several studies have found that virtual lesions in the PF area of the left supramarginal gyrus (SMG/PF), known to be involved in hand reenactment, did not impact the hand-tool compatibility task compared to the tool-tool compatibility task^2,14^. By contrast, in the hand-tool compatibility task, participants may solve the task with a direct matching between the structural characteristics of the tool and the shape of the hand (i.e., visual imagery/visuo-spatial skills). It would be particularly the case for “non-conflict” tools that are grasped to move or to use in the same way^22,23^. Indeed, the absence of competing effect in LBD patients for “non-conflict” tools may explain why the performance in the hand-tool compatibility tasks is similar to controls^15^.

Some studies found that virtual lesions made in the left SMG/PF impaired motor imagery (i.e., hand laterality judgment task) compared to virtual lesions made in left SPL which impaired visual imagery^24,25^. Thus, hand-tool compatibility tasks may call upon brain regions in the left tool processing network, as well as brain regions dedicated to the processing of visuo-spatial skills in bilateral SPL^24,26^, On the contrary, tool-tool compatibility tasks may be more left-lateralized in the tool processing network and do not engage more additional right brain regions than those classically observed in the right hemisphere for manipulation tasks (i.e., insula and pMTG^8^).

The aim of the present study is to explore the neurocognitive components engaged during the processing of manipulation knowledge, especially in tool-tool and hand-tool compatibility tasks. We conducted two controlled experiments in healthy participants and in brain-damaged patients. In the first experiment, we analyzed the impact of brain lesions on hand-tool and tool-tool compatibility tasks in the same experiment to examine the potential dissociations between these two tasks. We expect observing double dissociations between these two tasks, even if tool-tool compatibility task should be more impacted. In the second experiment, we aimed at exploring the cognitive bases of tool-tool and hand-tool compatibility tasks in healthy subjects. We hypothesize that both tasks rely upon common cognitive processes (i.e., manipulation knowledge), but also on distinct processes. We explore potential cognitive mechanisms by proposing that visual imagery/rotation may support hand-tool compatibility tasks, as this involves mentally rotating hands and tools as external objects to evaluate whether the shape of the hand matches the structural form of the tool. In contrast, motor imagery/rotation may underlie tool-tool compatibility tasks, requiring participants to imagine the position of their hand relative to the orientation of the tools to determine whether they would be manipulated in a similar way. In a third exploratory experiment, we reanalyzed fMRI data including tool-tool compatibility and hand-tool compatibility tasks, in which we hypothesis that both tasks may engage a common left-lateralized brain network within the tool processing network, but only the hand-tool compatibility task will be associated with activations in the right hemisphere, in brain regions known to be involved in visuo-spatial abilities.

## Experiment 1

### Participants

Sixty stroke patients (LBD: *n* = 30; RBD: *n* = 30) were recruited from the Neurological Vascular Unit at the CHU Minjoz in Besançon between March 2022 and June 2023. All patients came to the hospital to undergo a neurological and neuropsychological assessment approximately 2 months post-stroke (mean interval since lesion onset = 130.21 days, *SD* = 45.36; range = 71–296), as part of routine care. The patient-specific inclusion criterion was the presentation of a first-ever ischemic stroke in either the left or the right hemisphere. Patients with a history of psychosis, brain injury, drug, or alcohol abuse, or who were over 89 years old, were not included in the study. Normative data were obtained from a sample of 30 healthy subjects (mean age = 57.67 years, *SD* = 16.92, 19 females, 28 right-handers). Control participants were matched with LBD and RBD patients in terms of age, sex, and handedness. Montreal Cognitive Assessment scores (MoCA^27^) were higher for controls compared to LBD and RBD patients (all *ps* < 0.001). LBD and RBD patients were matched in terms of age, sex, handedness, interval since lesion onset, and MoCA scores, but not in terms of lesion volume.

Demographic data for each group of patients and for controls are displayed in Table 1.

**Table 1:** Demographic and clinical data of controls and brain-damaged patients

	LBD (*n* = 30)	RBD (*n* = 30)	Controls (*n* = 30)
Age (years)	62.40 (15.59)	62.53 (17.27)	59.17 (16.74)
Sex (Male/Female)	15/15	14/16	11/19
MOCA (/30)	**25.69** **(2.95)**^**a**^	**24.52** **(3.88)**	27.93 (1.74)
Handedness (Right/Left)	27/3	30/0	28/2
Lesion volume (cm^3^)	9.38 (24.07)	22.85 (32.81)	-
Interval lesion onset (days)	127.60 (38.87)	133.00 (52.01)^b^	-

Bold values indicated significant differences between patients and controls.

^a^MOCA scores were not available for one patient.

^b^Interval lesion onset days were not available for two patients.

Full written consent was obtained from all patients and controls. This study was conducted in accordance with the ethical standards of the Declaration of Helsinki and was approved by the local ethics committee of Bourgogne Franche-Comté University (CERUBFC-2022-02-15-006). All ethical regulations relevant to human research participants were followed.

### Methods

#### Materials and procedure

**Fig. 1:**
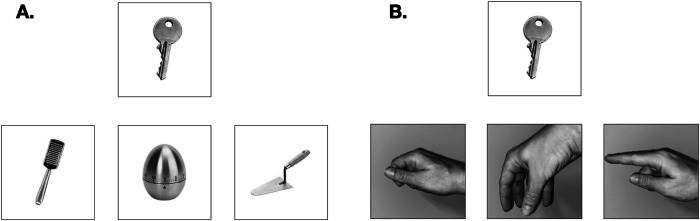
Example of items used in the action tool knowledge tasks in Experiment 1 and Experiment 2. **A** Tool-tool compatibility task. **B** Hand-tool compatibility task. Explanations are given in the text.

This experiment was made of two experimental tasks targeting action tool knowledge, that is, tool-tool compatibility and hand-tool compatibility tasks. Each of these tasks comprised 12 items and always began with two examples for which feedback on the correctness was given. Forty-eight greyscale pictures of familiar objects were used for this experiment. Each stimulus contains a target object (top) and three objects (bottom; for example, see Fig. 1). The list of all the items used in the experimental tasks is provided in Supplementary Table 1. All the participants completed the tasks in the same order (i.e., tool-tool compatibility and hand-tool compatibility tasks). Participants were given verbal cue to identify the object on the picture if they met difficulties to recognize it.

In the tool-tool compatibility task (Fig. 1A), participants had to choose among the three bottom familiar objects (e.g., *tap*, megaphone, kitchen scale), the one that is manipulated in the same manner as the target object (e.g., light bulb). An independent sample of 12 participants (*M*_*age*_ = 22.9, *SD*_*age*_ = 3.7, 8 females) was asked to rate each pair of objects (the target object and each of the three bottom familiar objects for a given item) by answering the question: *“Are these two tools manipulated in the same way when using them?”* (0: distinct manipulation, 5: similar manipulation). We found that for all items, the target pairs had a significantly higher rating in manipulation dimension compared to the two other pairs including the target object and the distractors (see Supplementary Table 1). In the hand-tool compatibility task (Fig. 1B), subjects had to choose among three pictures depicting a male hand posture the one that could fit with the tool (e.g., light bulb) in order to use it. All the familiar objects of the action tool compatibility tasks were presented before starting the experiment to ensure they were known (name and usage) by the participants.

Each correct answer given within 15 s was worth 1 point (maximum score = 12).

#### Imaging and lesion segmentation

Ischemic infarcts were delineated on acute phase diffusion-weighted imaging (DWI) sequences (TR 9190 ms, TE 88 ms, FOV 240 × 240 mm; 92 axial slices, flip angle 180◦). The lesions were identified and manually delineated on a slice-by-slice basis (GC). They were reviewed by a trained neurologist (EM) for accuracy in terms of lesion location and extent for each patient, providing cross examination by two examiners, as standard practice in manual delineation^28^. Binary masks were made from the lesions using ITK-Snap^29^. They were subsequently normalized to the MNI 152 space (1 mm resolution) using SMP12 (Wellcome Department of Cognitive Neurology, https://www.fil.ion.ucl.ac.uk/spm) for Matlab (MathWorks).

#### Data analysis

Correlational analyses (Spearman correlation coefficient *rho*) were conducted between chronological age and manipulation tasks in all groups. Moreover, we computed correlations between lesion volume and manipulation tasks in LBD and RBD patients.

We used non-parametric comparisons on raw scores between each group of patients and controls to test whether each group of patients presented a deficit in each task at the group level. Statistical results were corrected for multiple comparisons^30^. As controls did not perform equally in both conditions (i.e., hand-tool compatibility and tool-tool compatibility), patients’ raw scores were first transformed into z-scores to standardize the data with respect to the sample distribution. The z-score procedure involves subtracting the mean of the control group from a patient’s raw score and then dividing by the standard deviation of the control group, for each task and each patient. This transformation allowed for comparing patients’ performances independently of differences in the initial measurement scales and ensures a fair comparison between groups. Then, a group analysis was conducted using repeated measure analysis of variance (RM ANOVA) on patients’ z-scores, with Task (tool-tool compatibility *vs* hand-tool compatibility) as within-subject factor and Group as between-subject factor (LBD *vs* RBD).

For single-case analyses, for each patient, we performed two single Bayesian difference tests^31^. Each test quantified the degree to which the patient’s score on conceptual tool tasks dissociated from the control group using Bayesian methods to estimate the probability that a participant of the control group would obtain a lower score than the patient. We also reported the significant dissociations between tasks with the Revised Standardized Difference Test^32^. Finally, we examined the lesion location associated with neuropsychological dissociations by focusing only on patients showing greater impairment in hand-tool tasks compared to tool-tool tasks and on patients showing the reverse pattern in LBD and RBD patients.

### Results

#### Effect of clinical and demographical data on manipulation tasks

**Fig. 2:**
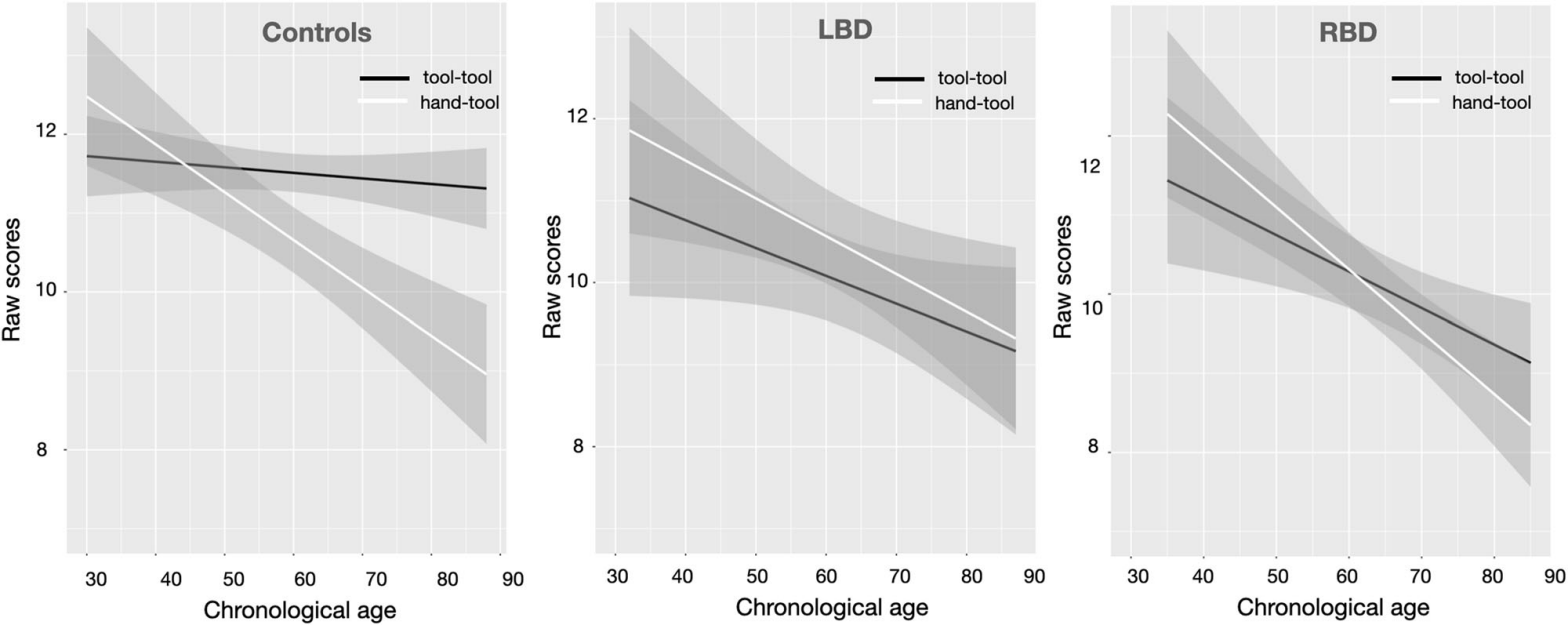
Correlation plots between chronological age and raw scores for controls and patients in tool-tool and hand-tool compatibility tasks. Linear regression lines and confidence regions are presented for each plot.

As it can be seen in Fig. 2, there was a significant association between chronological age and hand-tool compatibility raw scores in controls (*rho* = −0.71, *p* < 0.001), in LBD (*rho* = -0.40, *p* = 0.03) and in RBD (*rho* = −0.75, *p* < 0.001). There was no association between chronological age and tool-tool compatibility raw scores in controls (*rho* = −0.15, *p* = 0.44) and in LBD (*rho* = −0.33, *p* = 0.07), but there was one in RBD (*rho* = −0.53, *p* < 0.01).

We found that lesion volume was not associated with hand-tool and tool-tool raw scores in both groups of patients (all |*rho* | < 0.29, all *ps* > 0.12).

#### Group study

Considering raw scores, as controls performed better in the tool-tool compatibility task (*M* = 11.53, *SD* = 0.63) compared to the hand-tool compatibility task (*M* = 10.77, *SD* = 1.43, *p* = 0.009), a RM ANOVA was conducted on patient z-scores rather than raw scores. Whereas LBD (*M* = 10.00, *SD* = 0.28) and RBD (*M* = 10.10, *SD* = 0.23) performed worse in the tool-tool compatibility task compared to controls (all *Ws* > 164, all *ps* < 0.001), there was no difference between LBD (*M* = 10.33, *SD* = 0.33) and RBD (*M* = 9.8, *SD* = 0.36) compared to controls in the hand-tool compatibility task (all *Ws* > 304.5, all *ps* > 0.06).

**Fig. 3:**
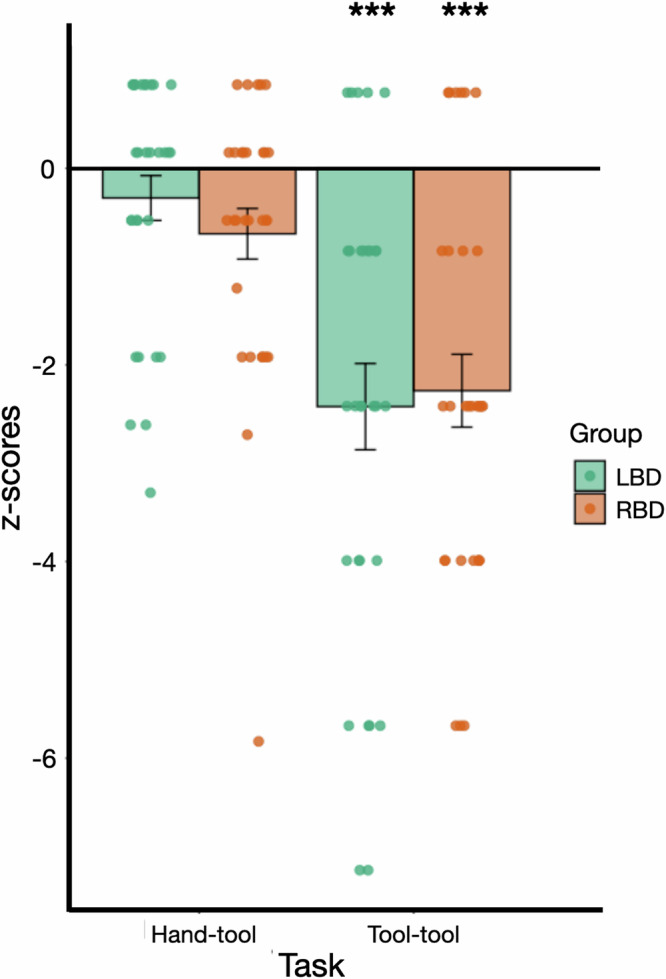
Effect of Task (tool-tool compatibility vs. hand-tool compatibility) and Group (LBD vs. RBD) on patients’ scores. Barplots represent mean z-scores, and error bars represent the Standard Error from the Mean (SEM). Significance (***p < .001) denotes corrected statistics for the comparisons between each patient group and the control group based on raw scores. Colored points (green: LBD and orange: RBD) represent individual z-scores.

The RM ANOVA was conducted on z-scores with Task (tool-tool compatibility *vs* hand-tool compatibility) as a within-subject factor and Group (LBD *vs* RBD) as a between-subject factor (see Fig. 3). As lesion volume was significantly different between LBD and RBD patients, this variable was included in the RM ANOVA as covariate. The RM ANOVA revealed a main effect of task, *F*(1,57) = 38.94, *p* < 0.001, *η*_*p*_^*2*^ = 0.41. Scores were lower in the tool-tool compatibility compared to the hand-tool compatibility task. There was no effect of Group, *F*(1,57) = 0.007, *p* = .93, *η*_*p*_^*2*^ = 0.00, nor an interaction between Task and Group, *F*(1,57) = 0.69, *p* = 0.41, *η*_*p*_^*2*^ = 0.01. There was no effect of lesion volume, *F*(1,57) = 2.16, *p* = 0.15, *η*_*p*_^*2*^ = 0.04, nor interaction between lesion volume and Task, *F*(1,57) = 0.26, *p* = 0.62, *η*_*p*_^*2*^ = 0.004.

#### Single-case analysis

The patient’s impairments and dissociations can be seen in Table 2. For the tool-tool compatibility task, 56.67% (*n* = 17/30) of LBD patients and 66.67% (*n* = 20/30) of RBD patients were impaired. For the hand-tool compatibility task, 23.33% (*n* = 7/30) of LBD patients and 26.67% (*n* = 8/30) of RBD patients were impaired. There were no significant differences in the proportion of LBD or RBD patients showing a deficit in the tool-tool compatibility task, *X*^2^(1) = 0.64, *p* = 0.43, or in the hand-tool compatibility task, *X*^2^(1) = 0.09, *p* = .77.

**Table 2:** Patients’ impairments and dissociations according to Crawford and Garthwaite’s (2005, 2007)

	Per cent of patients showing a deficit^a^		Per cent of dissociations^b^
	Hand-Tool	Tool-Tool	
LBD (%)	7/30 (23.33)	17/30 (56.67)	11/30 (36.67)
			HT > TT: 10/11
			TT > HT: 1/11
RBD (%)	8/30 (26.67)	20/30 (66.67)	10/30 (33.33)
			HT > TT: 9/10
			TT > HT: 1/10

*HT* Hand-Tool, *TT* Tool-Tool

^a^The presence of a deficit is attested according to the Crawford-Garthwaite Bayesian test for

single-case analysis (2007).

^b^The presence of a dissociation is attested according to the Revised Standardised Difference Test (Crawford and Garthwaite, 2005).

The proportion of dissociations between tool-tool compatibility and hand-tool compatibility was similar in LBD patients (36.67%, *n* = 11/30) and in RBD patients (33.33%, *n* = 10/30). Moreover, dissociations favoring better performance in hand-tool compatibility compared to tool-tool compatibility tended to be more frequent in both LBD (*n* = 10/11) and in RBD (*n* = 9/10) patients. Thus, while double dissociations were rare, they still occurred in both groups of patients.

#### Qualitative analysis of the lesion patterns and behavioral dissociations in hand-tool and tool-tool tasks

**Fig. 4:**
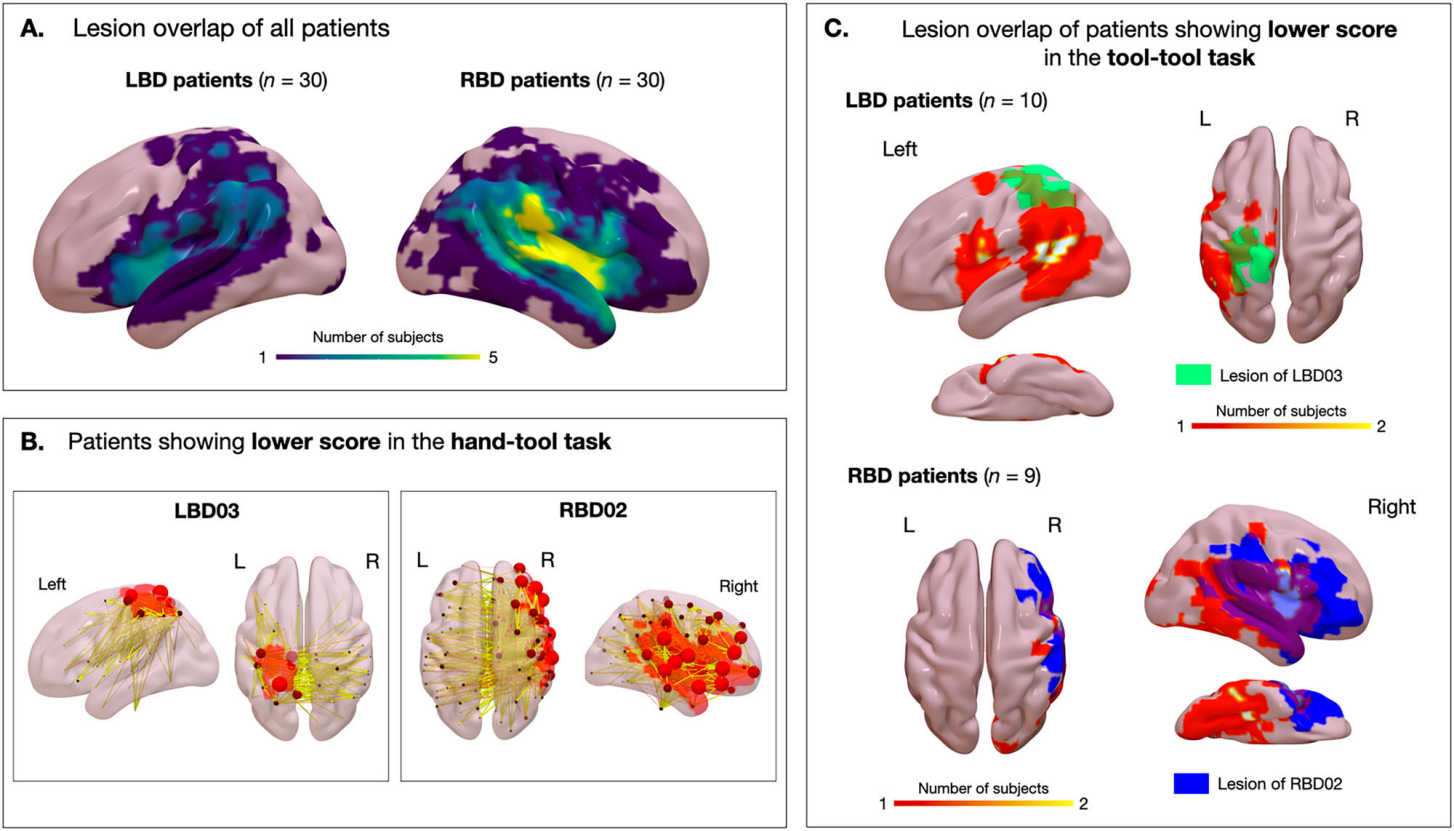
Analysis of lesions associated with dissociations between hand-tool and tool-tool tasks. The top left panel (**A**) shows the lesion overlap of all LBD and RBD patients. The bottom left panel (**B**) shows the lesion and disconnection patterns of LBD03 and RBD02 who showed lower scores in hand-tool task compared to tool-tool tasks. Nodes are sized and colored according to the total disconnection severity for each node. Edges are colored according to disconnection severity. Connectivity matrices were computed using the Schaefer et al. (2018) 100-region parcellation using the Lesion Quantification Toolkit (LQT; Griffis et al.^78^). The right panel (**C**) shows the lesion overlap of LBD and RBD patients showing the opposite pattern of behavioral dissociations. Lesions, disconnection severity (edges and nodes) and lesion overlap are projected on an MNI152 template present in the SurfIce viewer.

Interestingly, the lesion pattern associated with neuropsychological dissociation (see Fig. 4) revealed that LBD03 who scored lower in the hand-tool compared to the tool-tool task had lesion in the left superior parietal lobe and left post-central regions, with a disconnection pattern involving homologous regions in the right hemisphere. It appears that lesion overlap of LBD patients showing the reverse behavioral patterns may present more inferior within the left parietal lobe. RBD02 who scored lower in the hand-tool compared to the tool-tool task had an extensive right hemisphere lesion encompassing the frontal, temporal and parietal lobes, associated with several disconnections with the left hemisphere. Lesion overlap of RBD patients showing the reverse behavioral dissociations appear to be more restricted, particularly in frontal and parietal regions.

### Discussion

The purpose of this first experiment was to explore the impact of left and right brain lesions on manipulation knowledge tasks. We hypothesized that if hand-tool compatibility and tool-tool compatibility tasks rely upon separable processes, we should observe dissociations in brain-damaged patients.

We found that brain-damaged patients, regardless the side of the lesion, were more impaired in the tool-tool compatibility task compared to the hand-tool compatibility task, thus confirming in the same study, previous results found with distinct groups of LBD patients^15^. We also found that the frequency of dissociations was more significant in favor of the spared hand-tool compatibility task, indicating that tool-tool compatibility task is highly impacted by brain lesions^33–35^. However, the existence of double dissociations, even rare, may rule out the resource artifact effect and indicates that the two tasks are relying upon interrelated but separable processes. When exploring the lesion location, we found that a lesion in the left superior parietal lobe may cause greater impairment in hand-tool tasks than in too-tool tasks, whereas a large lesion in the right hemisphere, including the right parietal and right frontal regions may be associated with more severe hand-tool task impairment compared to too-tool task impairment. However, given the small sample, these results should be interpreted with caution.

Our results also highlight the sensitivity of the right hemisphere in tool-tool compatibility tasks, as suggested by group analysis and pattern of dissociations, which were highly similar in LBD and RBD patients. This result must be taken cautiously, as (1) the aim of this experiment was not to compare directly the two groups; and (2) the two groups were not matched in terms of lesion volume, although lesion volume entered as covariate in the group analysis did not modulate the difference between LBD and RBD patients. Moreover, the presence of apraxia was not tested in this study. Therefore, it is likely that including LBD apraxic patients would have potentially increased the differences found between LBD patients and controls in the present experiment^36^. In this case, we might also have observed a greater difference in LBD patients compared to RBD patients. In summary, these results suggest that the right hemisphere may also play a role in representing manipulation knowledge, as observed in studies using tasks partly supported by manipulation knowledge^37,38^, albeit potentially to a lesser extent than observed in LBD patients.

We also tested age-related effects on manipulation tasks. Whereas fMRI studies traditionally include young healthy subjects, controls matched with patients in brain damaged studies are generally older (fMRI mean age = 24.9 years old; LBD studies mean age = 62.2 years old^15^). We found that manipulation tasks were impacted distinctly, with hand-tool compatibility task being more sensitive to aging compared to tool-tool compatibility task. Similar findings have been previously observed in conceptual tool knowledge, with semantic tool knowledge being more impacted by aging compared to mechanical knowledge^39^. Interestingly, this result sheds a new light on previous data showing that LBD patients performed near from controls^9,10,34^. Indeed, we showed that controls were better in tool-tool compatibility task compared to compatibility hand tool task, whereas brain damage patients showed the opposite pattern. Brain lesions impact hand-tool compatibility but to a lesser extent than they do with the tool-tool compatibility task. Due to age-related effects, controls and brain damaged patients performed almost similarly, which suggest that brain damaged patients are using, in hand-tool compatibility task, cognitive processes that are distinct from those used in tool-tool compatibility task.

To sum up, these two tasks share similar brain networks, but they also call upon distinct neural and cognitive mechanisms. In a second experiment, we investigated some of the potential cognitive processes supporting these two tasks. We hypothesized that, compatibility tool-tool and hand-tool compatibility tasks are sharing core computation but also relies upon specific processes, that is motor imagery and visual imagery respectively.

## Experiment 2

### Participants

Fifty-two participants took part in Experiment 1 (*M*_*age*_ = 20.19, *SD*_*age*_ = 2.46, range = 18–32 years-old; 37 women). All participants were right-handers, had normal or corrected-to-normal visual acuity and reported no history of neurological or psychiatric disorder. All participants were voluntary and were naïve as to the purpose of the experiments. Written informed consent was collected from all participants in advance. The study was conducted in accordance with the ethical standards of the 1964 Declaration of Helsinki. All ethical regulations relevant to human research participants were followed.

### Methods

#### Materials and procedure

All the participants performed 5 tasks: action tool compatibility tasks (tool-tool and hand-tool tasks), motor imagery task, visual imagery task, and recognition of tool manipulation task. Programming the tasks and collecting the data were done with PsychoPy^40^, a cross-platform package running on the Python environment.

#### Action tool compatibility tasks

The tasks used in experiment 2 were computerized and slightly modified using PsychoPy^40^, and used in the present experiment to collect accuracies, as well as reaction times (RTs) (see Fig. 1). Half of the participants started with the tool-tool compatibility task and the other half with the hand-tool compatibility task. All the familiar objects of the action tool compatibility tasks were presented before starting the experiment to ensure they were known (name and usage) by the participants. The stimuli were presented twice, representing a total of 48 stimuli for both tasks (24 for Hand-Tool and 24 for Tool-Tool tasks). Participants answered by clicking with the mouse pad on the picture of the object or the hand corresponding to their choice. The mouse cursor appeared at the center of the screen (at equal distance from the object target and the pictures of hands or objects) at the same time as the stimulus was displayed and was removed as soon as the participant selected a picture. They were instructed to answer as fast as possible. Each trial starts with a fixation cross lasting for 500 ms followed by the stimulus that was presented as long as the participant had not selected an answer. As soon as the participant selected an answer, a white screen was displayed for 1 s, then the next trial was triggered. Accuracy (1 point was allowed for a correct answer, maximum score = 24 for each task) and RTs for correct answers were collected in this task.

#### Motor Imagery task

This task and stimuli were used in a previous study from our group^41,42^. Those stimuli were chosen because they represented four particular orientations of a hand with the thumb up, down, left and right. This hand was presented with either the palm or the back of the hand facing the participant. In sum, 16 pictures of hands were created (i.e., 4 orientations x 2 hand’s sides x 2 hands: Left and right) and presented four times in a row in a randomized order at each participant. The participant was instructed to respond as fast as possible whether the presented hand was a left or a right hand. He had the two index fingers resting on the keyboard (i.e., left index finger on the left of the keyboard and right index finger on the right) and responded with the left index finger when a left hand was presented or with the right index finger when a right hand was presented. Before each stimulus, a fixation cross was presented in the center of the screen during 500 ms followed by a white screen for 500 ms. As soon as the participant gave a response, the next stimulus was triggered. Accuracy (1 point was allowed for a correct answer, maximum score = 64) and RTs for correct answers were collected in this task.

#### Visual Imagery task

This task was used in a previous study from our group^41,43^. Two exemplars of a given figure were always presented on the computer screen. The exemplar presented on the right of the screen could be the exact copy of the exemplar of the left (i.e., identical) or could be the result of an axial symmetry relatively to the center of the screen (i.e., different). There were 16 identical and 16 different pairs of figures. The figure on the left was always presented on the same orientation while the figure on the right could be presented in four different orientations, corresponding to a clockwise rotation of 0°, 60°, 120°, or 180° from the original 0° orientation. The participant had to decide whether the two figures were identical or different by pressing, as fast as possible, the left key of the keyboard or the right key. Half of the participants had to answer ‘identical’ with the left key and the other half with the right key. Before each stimulus, a fixation cross was presented in the center of the screen during 500 ms followed by a white screen for 500 ms. As soon as the participant gave a response, the next stimulus was triggered. Accuracy (1 point was allowed for a correct answer, maximum score = 64) and RTs for correct answers were collected in this task.

#### Recognition of tool manipulation task

This task has been used in previous studies from our group^11,44,45^ and is one of the classical tasks to investigate the integrity of manipulation knowledge in patients suffering from apraxia. Participants are asked to select among several items the one that corresponded to the best way to hold a tool^46^. Ten familiar tool and their associated objects were used (hammer-nail, jug-glass, electrical plug-plug, match-matchbox, bottle opener-bottle, saw-piece of wood, scissors-twine, screwdriver-screw, key-keypad, light bulb-socket). In this test, we proposed four photographs, one with the correct male hand posture and three foils. Each photograph depicted a one-handed manipulation of the tool; the hold differed across photographs, but the relative position of the tools and objects did not vary. The foils were built according three conditions, that is, (1) Active part, the hand is located on the active part of the tool; (2) Uncomfortable hand posture, the hand is oriented on the tool in a way that its subsequent use will be uncomfortable; and (3) Impossible use, the position of the hand on the tool makes impossible its subsequent use. To sum up, there was 4 types of postures: one correct posture (Target), two uncomfortable but possible postures (Active part and Uncomfortable hand posture), and one impossible (Impossible use). Accuracy (1 point was allowed for a correct answer, maximum score = 10) and RTs for correct answers were collected in this task.

#### Data analysis

Accuracies and RTs were collected for each task. Correlational matrix (Pearson correlational coefficient *r*) with RTs corresponding to correct answers were calculated. To further explore the structural properties of the correlation matrix, it was subjected to multidimensional scaling which yields a graphical representation of the correlational structure^47^. Finally, to explain the variations in RTs in tool-tool compatibility and in hand-tool compatibility tasks, we carried out multiple regression analyzes, by considering the RTs in the Recognition Tool Manipulation (RTM) task, in Visual Imagery (VI) task and in Motor Imagery (MI) task as predictors. Multicollinearity between predictors was assessed by calculating the variance inflation factor (VIF). Outliers (i.e., influential observations) were identified with Cook’s distance and removed from the analysis.

### Results

#### Correlation between experimental tasks

Data (means and standard deviations) for each experimental task are displayed in Supplementary Table 2. Correlation matrix between dependent variables (Hand-Tool and Tool-Tool) and predictors variables are displayed in Table 3. We found that the two action tool tasks were positively correlated together (*r* = 0.53, *p* < 0.001). We also reported high significant correlations between the Recognition of Tool Manipulation task and the two action tool tasks (all *rs* > 0.53, all *ps* < 0.001), indicating that our experimental tasks are measuring action tool knowledge.

**Table 3:** Pearson correlations between dependent and independent variables of the experiment 2

	Hand-tool	Tool-tool	RTM	VI	MI
Hand-tool	-	0.53***	0.55***	0.63***	0.42*
Tool-tool		-	0.53***	0.50***	0.46***
RTM			-	0.31	0.28
VI				-	0.39*
MI					-

Correlations between dependent variables and predictors appear on grey background.

***p< 0.001, *p < 0.05.

*p-*values for correlations are adjusted for multiple testing.

*RTM*recognition of tool manipulation, *MI* motor imagery task, *VI* visual imagery task.

**Fig. 5:**
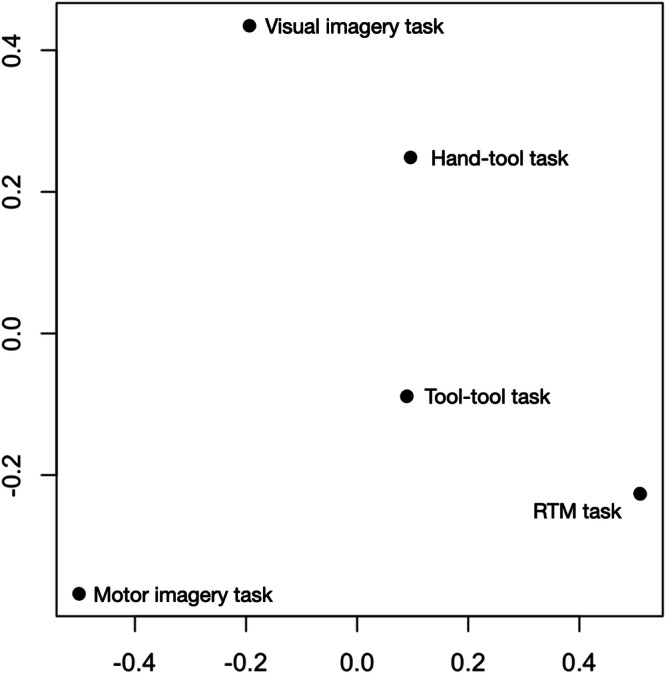
Multidimensional scaling of correlations between experimental tasks. RTM: recognition of tool manipulation. Explanations are given in the text.

To go further, we explored the structural properties of the correlation matrix, with multidimensional scaling. Higher correlations are represented by smaller distance between the respective data points. The distances correspond to the rank order of the correlations but not necessary to their absolute values. Multidimensional scaling analysis achieved a fair two-dimensional representation (Kruskal stress = 0.14, *r*^*2*^ = 0.93, see Fig. 5).

If the *x*-axis is difficult to interpret, it appears that Visual Imagery task and hand-tool compatibility task cluster towards the higher end of the *y*-axis, while Motor Imagery task, Recognition of Tool Manipulation task and tool-tool compatibility task cluster towards the lower end of the *y*-axis. This suggests that tasks sensitive to visual processes are distributed toward the upper part of the *y*-axis, whereas the tasks more sensitive to motor processes are distributed across the lower part of the *y*-axis.

#### Multiple regression analyses

To investigate which of the correlated variables explained a significant amount of variance of hand-tool compatibility and tool-tool compatibility tasks, we carried out multiple regression analyzes. Tests to see if the data met the assumption of collinearity indicated that multicollinearity was not a concern (VIF_*RTM*_ = 1.15, VIF_*VI*_ = 1.25, VIF_*MI*_ = 1.22). Outliers (i.e., influential observations) were identified with Cook’s distance and removed from the analysis.

When hand-tool compatibility reaction times were predicted, we found that RTM (*β* = 0.62, *p* < 0.001) and VI (*β* = 0.24, *p* = .035) were significant predictors, but not MI (*β* = 0.03, *p* = 0.79). the model was able to account for 46% of the variance, *F*(3,46) = 14.92, *p* < .001, *R*^*2*^ = 0.49, *R*^*2*^_*adj*_ = 0.46, *RMSE* = 1.024. When tool-tool compatibility reaction times were predicted, we found that RTM (*β* = 0.44, *p* = < 0.001) and MI (*β* = .31, *p* = .014) were found to be predictors, but not VI (*β* = 0.18, *p* = 0.13). The model was able to account for 52% of the variance, *F*(3,45) = 18.54, *p* < 0.001, *R*^*2*^ = 0.55, *R*^*2*^_*adj*_ = 0.52, *RMSE* = 1.028.

### Discussion

In this second experiment, we aimed at exploring the cognitive processes that support tool-tool compatibility and hand-tool compatibility tasks. We hypothesized that these tasks are heavily depending on manipulation knowledge which stores egocentric relationships^48^. We thus expected that both tasks may be explained by the RTM task, a task classically used to assess manipulation knowledge^9,44,45^. We also hypothesized that both tasks may rely upon additional specific processes, that is, visual imagery for the hand-tool compatibility task and motor imagery for the tool-tool compatibility task.

Our results showed that both hand-tool compatibility and tool-tool compatibility tasks are significantly predicted by the recognition of tool manipulation task, which is known to assess manipulation knowledge. To solve the recognition of tool manipulation task, participants need to distinguish the correct hand posture among several incorrect postures by activating the adequate hand-tool relationship in the context of use. The hand-tool compatibility task is quite similar as it is required to assess whether the hand posture may fit with a familiar tool. Thus, the hand grasp component appears important in these two tasks. Concerning the tool-tool compatibility task, the kinematics component appears more critical than the hand posture component because participants are asked to compare the motion made with two tools during familiar tool use. Hand posture and kinematics components are two interrelated components that are rarely dissociated in neurological patients^45,49,50^ and engaged similar posterior temporal and inferior parietal areas^3,6,8,13^.

As expected, we also reported that tool-tool compatibility task was predicted by the motor imagery task, whereas the hand-tool compatibility task was predicted by the visual imagery task. A motor imagery deficit is associated with a deficit of planning object-related actions in apraxic patients, which can explain why the motor imagery component may be related to the tool-tool compatibility task^51^. Concerning the hand-tool compatibility task, we proposed that this task can be solved without having a priori knowledge on hand-tool relationships, as deciding whether the configuration of the hand should fit with the structural properties of the tool may be sufficient. In our study, the link between tool use and hand posture were quite transparent but it may be possible that different results have come to us in case of conflict objects^22,23^.

Finally, these results also suggest that while the hand posture component is important in the hand-tool compatibility task, it relies more on visual rotation compared to motor rotation. Conversely, if the kinematics component is important in the tool-tool compatibility task, it relies more on motor rotation compared to visual rotation. However, the significant correlation observed between motor imagery and visual imagery tasks may suggest that the two tasks are not independent. Participants may be more likely to engage visual strategies in a given task without precluding that under other experimental conditions, motor strategies have been preferred. For instance, it has been shown that solving task in the first perspective (i.e., egocentric frame) involved motor activation^52^.

After demonstrating the common and some of the potential specific cognitive processes underlying hand-tool compatibility and tool-tool compatibility tasks in this second experiment, we now explored the neural correlates of these tasks by reanalyzing fMRI data. We hypothesize that both tasks may engage a common left-lateralized brain network within the tool processing network, but also distinct brain regions in left and right hemispheres.

## Experiment 3

### Participants

Thirty-four healthy subjects (*M*_*age*_ = 24.2, *SD* = 4.0, *range* = 18–36; 20 females; 34 right-handers) were enrolled in the study. Inclusion in the final sample required that head motion during scanning did not exceed 0.5 mm displacement (i.e., framewise displacement) between consecutive volumes on 90% of volumes, however, no participants were excluded based on this criterion. All participants had normal or corrected-to-normal vision and reported no history of neurological or psychiatric disorders. All participants were voluntary and signed written consent. The study was in line with the Declaration of Helsinki and was approved by the French Ethics Committee (N°ID-RCB: 2018-A00734-51). Subjects were paid for their participation. All ethical regulations relevant to human research participants were followed.

### Method

#### Stimuli and design

**Fig. 6:**
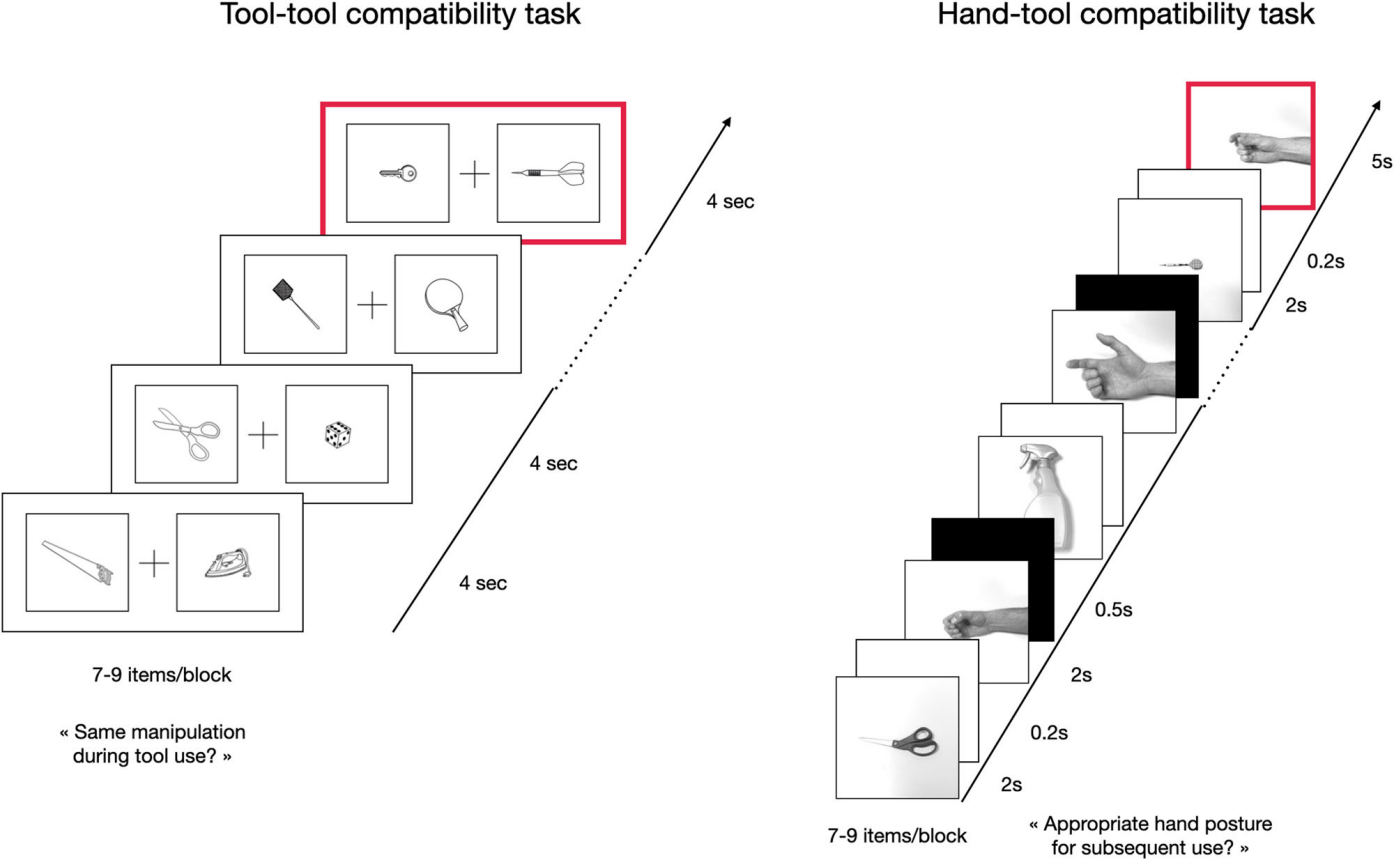
Example of stimuli and sequence of events used in the tool-tool compatibility task (left) and in the hand-tool compatibility task (right) in the experiment 3. Explanations are given in the text.

Each participant was scanned in a single fMRI session containing two functional runs. The first functional run (815 volumes, duration = 19 min) included the tool-tool compatibility task, followed by a second functional run containing the hand-tool compatibility task (1030 volumes, duration = 24 min). The compatibility tool-tool task has been described as Manipulation condition in a recent fMRI study, in which two other conditions were presented (i.e., Context and Function^8^), but not investigated in the present study. The session always started by the acquisition of an anatomical sequence lasting for about 8 min. Before entering the scanner, participants were familiarized with the experimental tasks. Practice trials were proposed for both tasks but did not re-appear in the fMRI experiment to avoid learning effects. An example of items used and the sequence of events in the fMRI experiment is provided in Fig. 6.

For the tool-tool compatibility task, a set of 27 black colored line-drawings of uni-manually manipulable tools (targets) were used. Participants saw the picture of the target tool presented with either a related item or an unrelated item. We created a set of 27 unrelated items and 27 related items. Thus, there was a total of 54 pairs of items, half related pairs and half unrelated pairs (see Supplementary Table 3). In each stimulus, the two items were surrounded by a black frame and appeared on either side of a black cross presented at the center of the screen. The target tool (e.g., saw) was associated either with a tool being manipulated in the same way (e.g., iron; related pair) or not (e.g., faucet; unrelated pair). Participants were asked to imagine if the two tools were manipulated in the same way. Here, participants did not have to focus on the hand posture but merely on the kinematic component. A control condition including 16 new pairs of unrelated items (Control_Tool-Tool; see Supplementary Table 4), was also constructed. No pairs used in the control condition were previously seen in the experimental condition. Each item pair of the control condition was prepared in such a way that half of the pair of items included at least one item with a significant black-colored surface (e.g., gun grip), which was not the case on the other half pair of items. Participants were asked to explore the items to find the presence or not of a black-colored surface on one of the two items. All pictures had a resolution of 960 × 720 pixels. A blocked within-subject design was used with alternating experimental blocks (duration = 24 s, 28 s or 32 s) and baseline periods (19 s). During each baseline period, participants were shown a white screen with a fixation cross. Items were blocked by condition (i.e., Tool-Tool and Control_Tool-Tool), with trials varying from 7 to 9 per block. Each stimulus duration was fixed and lasted for 4 s. There was no inter-stimulus interval. Six blocks were presented for Tool-Tool task and four blocks for the control condition, yielding a total of 10 blocks. Although each block varied from 24, to 28, to 32 s, after completing all 10 blocks, the average block length was similar for each condition (28 s). The number of related and unrelated pairs of stimuli was the same for each experimental condition and was balanced across all the blocks of the experiment. As a block contains 7, to, 8, to 9 items, related/unrelated pairs of stimuli could not be balanced within a block but were balanced across all the blocks of a given condition. There was the same number of items per condition. The order of the stimuli was randomized, while the order of blocks was pseudo-randomized to ensure that the experimental blocks were homogeneously distributed across the run. Participants were asked to answer only on the last trial of a block, indicated by a red frame surrounding the whole item. Participants indicated their response by pressing one of two buttons with the thumb of their right hand and indicated a ‘yes’ response with the left button and a ‘no’ response with the right button.

For the hand-tool compatibility task, a set of 48 items was created, each item consisting of a picture of a familiar tool (e.g., scissors, stapler, nutcracker, etc.) paired with a picture of a right male hand. The hand either match or not the tool’s proper usage (24 related and 24 unrelated pairs). The pictures of hands represented one of four prototypical gestures: precision grip, power grip, trigger, squeeze. Each tool was different whereas the images of the hands were drawn from the same set of four prototypical gestures throughout the experiment. Participants were asked to determine whether the picture of the hand posture was appropriate for using the tool. The control condition for the hand-tool compatibility task (Control_Hand-Tool) was composed of two sets of 24 pairs of items: either two tools or two hand postures. The two items could be the same or different. They were drawn from the Hand-Tool task, i.e., the 24 tools and the 4 prototypical gestures. In each set of pairs, the proportion of identical items was set to 50%. For the experimental and the control conditions, blocks could be 35.4 s (2 blocks), 40.1 s (2 blocks), or 44.8 s (2 blocks) long. Each block consisted of 7, 8, or 9 trials, and each trial in the hand-tool compatibility task started by the presentation of the image of a familiar tool for 2 s, then a white screen separated items for 0.2 s, then the image of a hand posture for the next 2 s, followed by a black screen separating trials for 0.5 s. For the last trial of each block, signaled with a red frame around the second picture, 3 additional seconds were given to allow for motor response. For the Control_Hand-Tool task, the two pictures could be either, with the same probability, two tools or two hand postures. In both conditions, trials had a 50% chance of being congruent trials (the tool can be used with this hand posture / the two images are the same). For all conditions, participants were instructed to perform mentally the task but to answer physically only for the last trial of each block, indicated by a red frame surrounding the whole item. The answer had to be given by pushing with the thumb on one of the two buttons of an fMRI-compatible button box.

For the tool-tool compatibility task, 25 participants (*M*_*age*_ = 26.6, *SD*_*age*_ = 7.4, 18 females) who were not enrolled in the fMRI experiment were asked to classify the 54 pairs of stimuli based on whether they were manipulated in the same way. Participants correctly classified the pairs with an average accuracy of *M* = 84.07% (*SD* = 15.26). For the hand-tool compatibility task, 15 participants (*M*_*age*_ = 23.00, *SD*_*age*_ = 3.78, all female), also not involved in the fMRI experiment, were asked to classify each pair of items by determining whether each hand posture was appropriate for using the tool. Participants correctly classified the pairs with an average accuracy of *M* = 81.39% (*SD* = 5.37). Furthermore, there was no difference between the scores obtained in the two tasks, *t*(31.84) = 0.92, *p* = 0.37 (two sample Welch *t*-test).

For both tasks, participants were advised to remain attentive all through the block and to perform the task even if no response was expected. As blocks did not have equal lengths, participants had to stay awake and alert inside the scanner as they were expected to answer the last trial of each block but had no means of predicting the end of this block. This strategy allowed us to ensure that participants remained focused on the task while avoiding confounding effects of motor execution. The last item of each block (which was surrounded by the red frame), for which a motor response was produced, was not included in the analysis. In the scanner, stimuli were back-projected onto a screen (60 Hz frame rate, 1024 × 768 pixels screen resolution) via a LCD projector (LX 501, CHRISTIE) and viewed through a mirror mounted on the head coil. Images on the screen had dimensions of 36 × 24 cm.

#### fMRI data acquisition

Imaging data were acquired on a 3 T Siemens Prisma Scanner (Siemens, Erlangen, Germany) using a 64-channel head coil. Blood-Oxygen Level Dependent (BOLD) images were recorded with T2*-weighted echo-planar images (EPI) acquired with the multi-band sequence. Functional images were all collected as oblique-axial scans aligned with the anterior commissure–posterior commissure (AC–PC) line with the following parameters: 815 volumes per run, 57 slices, TR/TE = 1400 ms / 30 ms, flip angle = 70°, field of view = 96 × 96 mm^2^, slice thickness = 2.3 mm, voxel size = 2.3 × 2.3 × 2.3  mm^3^, multiband factor = 2. Structural T1-weighted images were collected using an MPRAGE sequence (224 sagittal slices, TR/TE = 3000 / 2.93 ms, inversion time = 1100 ms, flip angle = 8°, 224 × 256 mm FOV, slice thickness = 0.8 mm, voxel size = 0.8 × 0.8  × 0.8 mm^3^).

#### Preprocessing of fMRI data

Structural T1-weighted images were segmented into tissue type (GM: grey matter, WM: white matter and CSF: cerebro-spinal fluid tissues) using the Computational Anatomy Toolbox (CAT12; http://dbm.neuro.uni-jena.de/cat12/) segmentation tool, in order to facilitate the normalization step. Functional data were analyzed using SPM12 (Wellcome Department of Cognitive Neurology, http://www.fil.ion.ucl.ac.uk/spm) implemented in MATLAB (Mathworks, Sherborn, MA). The first 3 EPI volumes were collected and discarded prior to the start by the scanner to allow for T1 equilibration effects. No slice-timing correction was applied. Preprocessing for univariate analyses included the following steps (1) 3D motion correction and linear detrending; (2) realignment to the mean EPI image with 6-head motion correction parameters and unwarping using topup^53^; (3) co-registration of the individual functional and anatomical images; (4) normalization towards MNI template; and (5) spatial smoothing of functional images (Gaussian kernel with 5 mm FWHM).

#### GLM analysis

A general linear model was created using design matrices containing one regressor (explanatory variable) for each condition (i.e., Tool-Tool, Hand-Tool, Control_Tool-Tool, Control_Hand-tool) modeled as a boxcar function (with onsets and durations corresponding to the start of each stimulus of that condition) convolved with the canonical hemodynamic response function (HRF) as well as its temporal and derivatives dispersion. Six regressors of non-interest resulting from 3D head motion estimation (x, y, z translation and three axis of rotation) were added in the design matrix. The model was estimated in each participant, also taking into account the average signal in the run. After model estimation, we computed the two simple contrasts at the first level (i.e., experimental conditions against control conditions) that were transferred to a second level group analysis (one sample *t-*tests) to obtain the brain regions activated in the Tool-Tool task (Tool-Tool > Control_Tool-Tool) and in the Hand-Tool task (Hand-Tool > Control_Hand-Tool). Second, we calculated the contrasts Hand-Tool > Tool-tool and Tool-Tool > Hand-Tool contrasts, to identify the brain regions where BOLD activity significantly differed across the two conditions. We present results maps with a cluster-forming threshold set at *p* = 0.001 and a cluster-level threshold set at *p* < 0.05, corrected for family-wise error (FWE).

## Results

**Fig. 7:**
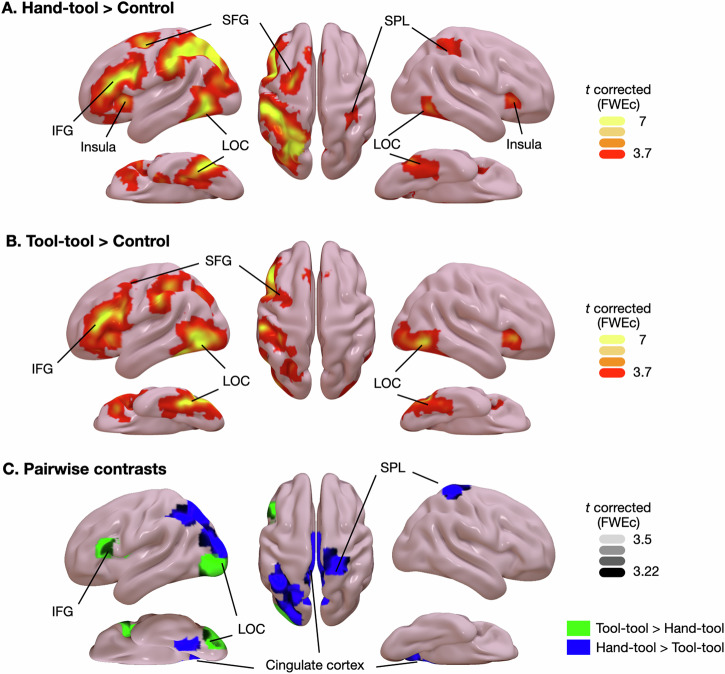
Statistical maps for the contrasts. **A** Hand-Tool > Control (threshold of spatial extent = 85), **B** Tool-Tool > Control (threshold of spatial extent = 185), (**C**) Hand-Tool > Tool-Tool (threshold of spatial extent = 70) and Tool-Tool > Hand-Tool (threshold of spatial extent = 76) are projected on an MNI template and are FWE-corrected (*p* < 0.05) for multiple comparisons across the whole-brain at the cluster level. IFG: inferior frontal gyrus; LOC: lateral occipitotemporal cortex; SFG: superior frontal gyrus; SPL: superior parietal lobe.

We first computed the simple contrasts Hand-Tool>Control and Tool-Tool>Control (see Fig. 7 and Table 4). Contrasting Hand-Tool task and Tool-Tool task with their respective control conditions revealed activations in a widespread bilateral brain network, corresponding to the tool processing network, therefore mainly lateralized in the left hemisphere (see Fig. 7A, B). We found activations in bilateral insula, bilateral lateral occipital temporal cortex, left inferior frontal gyrus, left supramarginal gyrus/intraparietal sulcus, and left superior frontal gyrus. The main contrast also revealed the activation of an additional region located in the right superior parietal lobe for the Hand-Tool task only. When contrasting Hand-Tool against Tool-Tool tasks, we found activations in left TOFc/LOTC, left pIPS, bilateral cingulate cortex, and right SPL (see Fig. 7C). When contrasting Tool-Tool against Hand-Tool tasks, we found activations in left IFG and left LOC.

**Table 4:** Local maxima of activation clusters (MNI stereotactic coordinates) for the individual contrasts Hand-Tool> Control, Tool-Tool>Control, Hand-Tool>Tool-Tool, Tool-Tool>Hand-Tool

Brain region	Hemisphere	Peak MNI coordinates			Cluster size	*T*-value	*P*FWE
*Hand-Tool > Control*							
LOC	Left	−27	−71	36	5478	12.00	<0.001
IFG *po*	Left	−41	16	25	1911	10.11	<0.001
SFG	Left	−22	−9	57	546	8.59	<0.001
Insular cortex	Left	−27	23	2	185	8.33	<0.001
Cerebellum VIIb	Right	19	−73	−49	745	8.32	<0.001
Cerebellum IX	Right	14	−55	−47	254	7.37	<0.001
SFG/Paracingulate cortex	Left	−6	23	48	226	7.20	<0.001
Insular cortex	Right	33	23	−1	85	6.32	0.025
ITG/LOC	Right	46	−60	−8	381	5.99	<0.001
Postcentral gyrus	Right	35	−32	45	143	5.28	0.002
Intracalcarine cortex	Right	17	−64	8	248	4.93	<0.001
Thalamus	Left	−15	−30	6	106	4.73	0.009
*Tool-tool > control*							
IFG *pt*	Left	−45	27	18	2316	10.83	<0.001
FG / SMG / TOFc	Left	−38	−55	−12	3661	9.36	<0.001
Cerebellum VIIb	Right	19	−76	−49	2484	8.95	<0.001
SFG/Paracingulate cortex	Left	−4	21	48	448	8.07	<0.001
FOc/Insular cortex	Right	33	25	−3	185	6.70	<0.001
*Hand-Tool > Tool-Tool*							
Cingulate gyrus	Left	−4	−25	29	341	6.08	<0.001
Precuneus	Left	−11	−64	27	459	6.06	<0.001
LOC	Left	−20	−66	48	298	5.70	<0.001
TOFc	Left	−31	−48	−8	79	5.03	0.03
Precuneus	Left	−4	−48	50	224	4.91	<0.001
Occipital Pole	Left	−22	−94	15	157	4.43	0.001
SPL/Postcentral gyrus	Right	21	−44	71	70	4.21	0.049
*Tool-Tool > Hand-Tool*							
LOC	Left	−38	−85	−3	148	5.23	0.001
IFG *pt*	Left	−41	23	15	76	4.70	0.035

All results are thresholded at *p* < 0.05 (FWE, cluster level).

Brain region labels are given according the Harvard-Oxford Cortical and Subcortical Structural Atlas.

*LOC* lateral occipital cortex, *IFG po* inferior frontal gyrus pars opercularis; *SFG* superior frontal gyrus; *ITG* inferior temporal gyrus; *IFG pt* inferior frontal gyrus pars triangularis; *FG* fusiform gyrus; *TOFc* temporo-occipital fusiform cortex; *FOc* frontal orbital cortex; *SPL* superior parietal lobe.

We also contrasted the control conditions between each other (see Supplementary Fig. 1 and Supplementary Table 5). Contrasting Control_Hand-Tool with Control_Tool-Tool revealed a right brain activation in SMG/MTG, whereas contrasting Control_Tool-Tool with Control_Hand-Tool led to significant activations in bilateral temporal occipital fusiform cortex, and left precuneus.

## Discussion

We found that both conditions engaged similar brain regions mainly lateralized in the left hemisphere, that is, IFG, LOTC and SMG/IPS. These results are in line with previous studies showing the involvement of these brain regions for either tool-tool or/and hand-tool compatibility tasks^3,5,6,8,13,15^. Hand-tool compatibility and tool-tool compatibility tasks also engaged brain regions in the right hemisphere, i.e., the LOTC which is coactivated with the left LOTC in action tool knowledge tasks^54^. These results confirm that these two tasks traditionally used to assess manipulation knowledge are calling upon similar brain regions, known to be part of the tool processing network.

The analyzes also revealed some differences when contrasting hand-tool and tool-tool compatibility tasks. For instance, the left pIPS was more engaged in the hand-tool compatibility task compared to the tool-tool compatibility task. However, when contrasting both control conditions, we found that the control condition associated with tool-tool compatibility elicited more brain activity in the left pIPS than the control condition associated with Hand-tool compatibility task. This result suggests that the difference in the left pIPS is rather due to control conditions difference than true differences between hand-tool and tool-tool compatibility tasks in the posterior parietal cortex. We also found the involvement of left SPL in both conditions, but only in the right SPL for the hand-tool compatibility task. The right parietal lobe has been associated with various cognitive processes, that is visuospatial skills and visual imagery^24,26^. In a rTMS study, it has been found that a virtual stimulation in left and right SPL impairs the mental rotation of a letter (visual imagery), whereas the virtual stimulation made in left and right SMG impairs the mental rotation of a hand (motor imagery)^24^. We also found more engagement of the bilateral posterior cingulate cortex in the Hand-Tool task, a large region belonging to the default mode network, and involved in regulating the focus of attention^55^. Further studies are needed to better understand the role of this region in hand-tool and tool-tool compatibility tasks.

Contrasting tool-tool compatibility task with hand-tool compatibility task yield significant activation in left IFG and left posterior LOTC. In broad terms, left IFG and left LOTC were more involved in tool-tool compatibility task than in hand-tool compatibility task. The LOTC plays a role in the perception of object-related actions^56–58^ and the conceptual processing of tools and actions^59–61^. LOTC also includes hand-selective representations that closely overlap with regions activated by tools^62,63^, suggesting that hand and object representations were tightly integrated during the tool-tool compatibility task. IFG was identified in tool use production and understanding^64,65^, and may provide a top-down biasing signal that resolve competition between candidate tool use actions^17,22,23^. One may assume that the selection of actions associated with the two tools in the Tool-Tool condition may potentiate the top-down regulation of left IFG compared to hand-tool compatibility task.

If a virtual lesion within the left SMG (SMG/PF) had a greater impact on the tool-tool compatibility task compared to the hand-tool compatibility task^2,14^, our findings suggested that brain activity within left SMG was similar between the hand-tool and tool-tool compatibility tasks. These results collectively indicate that the presence of a lesion within the left SMG alone may not suffice to produce a deficit in the hand-tool compatibility task, as additional cognitive processes, supported by other brain regions, may be recruited to compensate for this task.

The tasks used in Experiment 3 differ in several ways from those in Experiment 1 and 2, raising the question of whether the neural correlates would have been the same across these experiments. The primary difference lies in the presence of distractors, which were included in Experiment 1 and 2 but not in Experiment 3. While it is clear that the core neurocognitive mechanisms underlying manipulation knowledge are engaged in all three experiments, one could hypothesize that the inclusion of distractors in Experiment 3 have modulated the BOLD response by influencing the salience of the target depending on the nature of the distractors. Furthermore, the presence of distractors might also have impacted the reliance on visual and motor imagery. This experiment represents a preliminary step, suggesting that the neural correlates may differ between hand-tool and tool-tool tasks. However, additional studies are required to validate this exploratory finding.

### General discussion

In this study, we explored the neurocognitive bases of two tasks classically used to assess action tool knowledge, that is, tool-tool and hand-tool compatibility tasks. Although these tasks are used interchangeably in the literature^3,13^, the performance obtained in these tasks can vary considerably according to neuropsychological data^9,10,33–35^, therefore suggesting that they rely upon, at least in part, distinct mechanisms. Our results are in general agreement with this hypothesis. We will now discuss in turn the main findings of the present study.

We found that tool-tool and hand-tool compatibility tasks are highly related with the recognition of tool manipulation task, thus indicating that these both tasks assess manipulation knowledge. Moreover, these two tasks engage similarly several part of the tool processing network, notably the left SMG, part of the ventro-dorsal pathway, known to support abstract representations about action tool knowledge^2,8,13,15,20,21,66,67^. The left SMG may be considered as a ventro-dorsal hub interacting with proximal and distal parts of the tool processing network in order to integrate several representations about tool use^8,62^. Other brain regions were found to be associated with these two tasks in the right hemisphere, that is, the right LOTC. During action observation the right LOTC is able to decode abstract representation about transitive actions^56,57,68^ and is often activated during action tool knowledge tasks but is rarely studied in brain damaged patients using large sample of LBD patients^64,69,70^. However, some studies found that the right pMTG may play a role in transitive actions^37,38^. Further studies are needed to explore the role of this brain region in action tool knowledge and the interaction between left and right pMTG during the retrieval of action tool knowledge.

Beyond the similarities of these two tasks, we also reported several differences. Although they both engaged the same pattern of brain regions in the tool processing network, some brain regions were more engaged in one or the other task within, but also outside the tool processing network. For instance, we found that the left IFG was more engaged in the tool-tool compatibility task whereas superior part of the left LOTC was more engaged in the hand-tool compatibility task, suggesting that if both tasks rely upon the left tool processing network, they also engage in a specific way the distinct brain regions involved in the tool processing network. The involvement of the left IFG in the tool-tool task aligns with the idea that this task requires the activation and comparison of gesture engrams associated with the use of the presented tools. The IFG may be a suitable candidate for this process, as it has been associated with both working memory and the understanding and execution of actions^8,65,71^. Moreover, it is noteworthy that only the hand-tool task engaged the right SPL, a brain region outside the tool processing network, which is known to be involved in visuo-spatial skills and visual imagery^24,26^. Thus, hand-tool task may engage brain regions associated visual strategies that tool-tool task did not. However, we can draw only indirect conclusions from the fMRI experiment (Experiment 3) and the results obtained from healthy participants (Experiment 2). Therefore, further studies are needed to confirm the engagement of this brain structure in the hand-tool task. The presence of neuropsychological dissociations between the two tasks in both LBD and RBD patients confirmed the specificity of each task even if the presence of impaired hand-tool performance in presence of spared tool-tool was rare compared to the opposite pattern. Moreover, the pattern of dissociations was similar in both LBD and RBD patients, ruling out the fact that hand-tool compatibility and tool-tool compatibility tasks may be supported by specific brain hemispheres. However, the patients included in this study had lower lesion volume compared to similar other studies^64,69^, and RBD patients had higher lesion volume than LBD patients, thus it may be possible that the impairments of LBD patients were underestimated compared to RBD patients.

When considering the potential cognitive processes underlying hand-tool and tool-tool compatibility tasks, we found that they were explained by visual and motor imagery, respectively. It has been reported that independent of the stimulus to be rotated, RBD patients were found to be selectively impaired in performing operations based on the reference frame of the object (i.e., “visual strategy”), but still able to perform viewer-based mental rotation (i.e., “motor strategy”). By contrast, LBD patients were found to be selectively impaired in mental rotation based on the viewer’s reference frame, leaving object-based mental rotation intact^72^. This suggests that mental rotation is achieved by recruiting different strategies, each sustained by a unilateral brain network. Taken together, considering that manipulation knowledge is left-lateralized in the brain, tool-tool compatibility task should have been impaired in LBD but not in RDB patients while hand-tool compatibility task would have been spared in both groups. However, this was not the case. As tool-tool compatibility task was impaired in both group of patients, one may assume that manipulation knowledge may be supported bilaterally in the brain^45^, however, it could not explain the pattern obtained with the hand-tool compatibility task. If visual and motor imagery are dissociated processes^52^, visual imagery was impacted following bilateral SPL stimulation and motor imagery following bilateral SMG stimulation^24^, thus questioning a pure functional asymmetry between visual and motor imagery processes. Moreover, the hand laterality judgement task, as we used in our study, engaged the right SPL and SPL is known to contain postural representations of the upper limb^73^, suggesting that both motor and visual rotation share neural computations and it is therefore likely that more complex interactions exist between these two processes and their neural correlates.

One important point raised in the present study is the distinct age-related effects observed in manipulation tasks, with tool-tool compatibility task being less impacted by aging compared to hand-tool compatibility task. This is of first importance as we explored the neurocognitive bases of manipulation tasks in healthy young subjects in the fMRI (Experiment 1) and behavioral experiment (Experiment 3) whereas the controls were older in Experiment 2^74,75^. One should be careful when comparing healthy young and healthy elderly, as aging may modify the neurocognitive bases and/or strategies engaged in a given function^39^. For instance, the tool processing network may not be functionally reorganized with aging during a tool use task^76^. However, a switch in strategical procedure implying a stronger involvement in planning and preparatory phase of transitive actions was observed in elderly. It also applies on visual and motor imagery strategies which change with aging^76,77^. Thus, further studies may be needed to assess whether the same predictors explained the variance in manipulation tasks between young and older healthy subjects. Finally, the smaller difference observed between controls and LBD patients in hand-tool compatibility compared to tool-tool compatibility tasks^15^ may also be explained by a decrease in controls’ performance.

## Conclusions

In the present work, we found that two tasks classically used to assess the integrity of manipulation knowledge, that is hand-tool compatibility and tool-tool compatibility tasks rely upon similar but also distinct neurocognitive processes. Our results had several implications on clinical investigations of manipulation knowledge, by questioning the tasks used and their cognitive bases. Future directions should now directly explore the link between manipulation tasks and visual and motor imagery strategies, by also considering age-related effects.

### Reporting summary

Further information on research design is available in the Nature Portfolio Reporting Summary linked to this article.

## Supplementary information

Supplementary material

Reporting Summary

## Data Availability

The data that support the findings of this study are openly available in OSF at https://osf.io/yh7tm/.

## Code availability

The codes used in this study are available upon reasonable request.
