# Supplementary material for: Neurocognitive mechanisms underlying action tool knowledge tasks: specificity of tool-tool compared to hand-tool compatibility tasks
Source: Commun Biol. 2025 Apr 3;8:552. doi: 10.1038/s42003-025-07923-1 (PMC11968931; doi:10.1038/s42003-025-07923-1)
Supplement: Supplementary file 1 — Supplementary material [file 42003_2025_7923_MOESM1_ESM.pdf]

Lesourd, Osiurak, Martin, Hague, Laroze, Clément, Medeiros de Bustos, Fargeix, Magnin and Moulin

Neurocognitive mechanisms underlying action tool knowledge tasks: specificity of tool-tool compared to hand-tool compatibility tasks

Supplementary material

Supplementary table 1. Object pairs (related and unrelated) used in the tool-tool compatibility task (Experiment 1)

corkscrew	*dart*	***spinning top***	*spray*
bank card	***plug***	*stamp*	*highlighter*
light bulb	**tap**<br>3.75 (.87)	megaphone<br>1.17 (.39)	kitchen scale<br>1.08 (.29)
key	grater<br>1.33 (.65)	**timer**<br>4.33 (.65)	trowel<br>1.58 (.90)
saw	door handle<br>1.33 (.65)	padlock<br>1.67 (1.07)	**scraper**<br>3.75 (.87)
compass	USB key<br>2.33 (.98)	**lemon squeezer**<br>3.83 (1.03)	hand gun<br>1.17 (.39)
hammer	bolt<br>1.58 (.67)	lighter<br>1.42 (.67)	**drumstick**<br>3.33 (.98)
eyedropper	suitcase<br>1.08 (.29)	hanger<br>1.17 (.39)	**tongs**<br>4.08 (1.00)
clothespin	**shears**<br>4.17 (.58)	candle<br>1.33 (.65)	coffee machine<br>1.25 (.62)
screwdriver	**timer**<br>4.08 (.67)	scissors<br>1.67 (1.15)	Christmas ball<br>1.17 (.39)
coffee maker	plectrum<br>1.25 (.45)	**measuring glass**<br>**4.75 (.45)**	golf ball<br>1.33 (.49)
meat grinder	wall stapler<br>2.25 (.62)	**music box**<br>**4.58 (.51)**	drill<br>1.50 (.52)
hand grenade	**paper plane**<br>**3.00 (.85)**	jug<br>1.08 (.29)	chopper<br>1.08 (.29)
horn	protractor<br>1.17 (.39)	rake<br>1.33 (.89)	**stapler**<br>**4.58 (.51)**

Italic items were used as examples
Target items appear on the left whereas associated items are represented in bold
Values represent mean ratings and standard deviations of the mean for the manipulation dimension
Ratings for the related pairs were significantly higher than for the unrelated pairs (all *ps* $< .0001$)

Lesourd, Osiurak, Martin, Hague, Laroze, Clément, Medeiros de Bustos, Fargeix, Magnin and Moulin

Neurocognitive mechanisms underlying action tool knowledge tasks: specificity of tool-tool compared to hand-tool compatibility tasks

Supplementary table 2. Means and standard deviations for each task of Experiment 2

	Accuracy (%)		RTs (ms)	
	mean	*sd*	*mean*	*sd*
Hand-Tool	88	9	4038	194
Tool-Tool	79	15	4444	153
RTM	94	9	3999	135
VI	87	18	2598	123
MI	89	12	2194	913

RTM: recognition of tool manipulation; MI: motor imagery task; VI: visual imagery task

Supplementary table 3. Object pairs (related and unrelated) used in the tool-tool compatibility task (Experiment 3)

Target objects	related	unrelated
bottle opener	water fountain	sword
hand vacuum	paint roller	hammer
squeeggee	tennis racket	nutcracker
knife	spatula	mouse computer
calculator	switch	salt shaker
match	brush	dumbbells
saw	iron	tap
rub	nail file	chess king
guitar	fan	syringe
rattle	lasso	snap hook
stapler	nail clippers	chalk
screwdriver	nutcracker	pan
light bulb	corkscrew	pitcher
gun	spray bottle	petanque balls
chopper	fishing rod	shears
scissors	garlic press	dice
pickaxe	whip	watering can
wrench	doorknob	bucket
fly swatter	pingpong racket	boomerang
tin can	grenade	needle
masher	ski stick	feather duster
pepper mill	rotating hose reel	plastic bag
key	timer	dart
plug	credit card	spinning top
kitchen whisk	salad spinner	trowel
clothespin	staple remover	measuring tape
rake	peeling knife	ladle

Supplementary table 4. Object pairs used in the control condition of the tool-tool task (Experiment 3)

colored black surface pairs		no colored black surface pairs	
wrench	sponge	pickaxe	corkscrew
fly swatter	push mower	key	chainsaw
tin can	slicing machine	kitchen whisk	synthesizer
masher	laptop computer	clothespin	triangle
pepper mill	lighter	jackhammer	drill
plug	eraser	mosquito net	saber
rake	paper clip	potato ricer	cutter
icebox	meat grinder	whip	flashlight

Objects that contain a black colored surface are underlined

Lesourd, Osiurak, Martin, Hague, Laroze, Clément, Medeiros de Bustos, Fargeix, Magnin and Moulin

Neurocognitive mechanisms underlying action tool knowledge tasks: specificity of tool-tool compared to hand-tool compatibility tasks

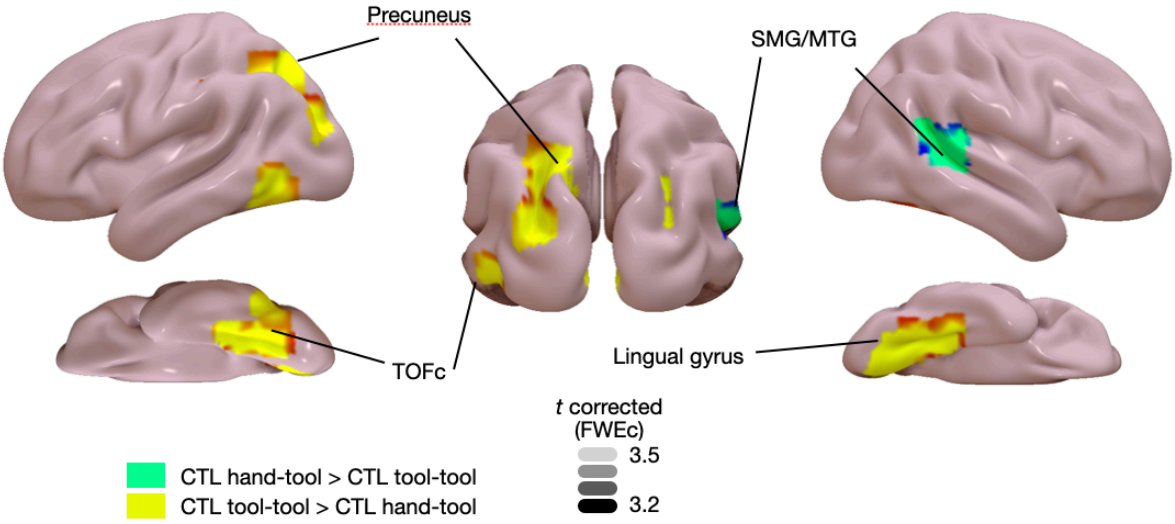

Supplementary figure 1. Statistical maps for the comparison between control conditions (Experiment 3). Control_Hand-Tool > Control_Tool-Tool and Control_Tool-Tool > Control_Hand-Tool contrasts are projected on MNI cortical surface and are FWE-corrected ($p < .05$) for multiple comparisons across the whole-brain at the cluster level. SMG: supramarginal gyrus; MTG: middle temporal gyrus; TOFc: temporal occipital fusiform cortex.

Supplementary table 5. Local maxima of activation clusters (MNI stereotactic coordinates) for the individual contrasts between control conditions (Experiment 3)

Brain region	Hemisphere	Peak MNI coordinates			Cluster size	T-value	*P* FWE
		x	y	z			
CTL Hand-Tool > CTL Tool-Tool							
SMG/MTG	Right	51	-39	11	74	4.64	.029
CTL Tool-Tool > CTL Hand-Tool							
Cerebellar Vermis VI	Left/Right	1	-71	-26	103	8.15	<.001
Lingual gyrus	Right	10	-69	-5	601	7.38	<.001
TOFc	Left	-29	-50	-10	195	5.50	<.001
Precuneus	Left	-22	-60	31	412	5.49	<.001
Cerebellar X	Right	26	-37	-47	84	5.29	.007
SMG	Left	-36	-37	34	66	4.07	.017

All results are thresholded at $p < .05$ (FWE, cluster level)
Brain region labels are given according the Harvard-Oxford Cortical and Subcortical Structural Atlas
SMG: supramarginal gyrus; MTG: middle temporal gyrus; TOFc: temporo-occipital fusiform cortex;
