# Supplementary material for: Neurocognitive mechanisms underlying action tool knowledge tasks: specificity of tool-tool compared to hand-tool compatibility tasks
Source: Commun Biol. 2025 Apr 3;8:552. doi: 10.1038/s42003-025-07923-1 (PMC11968931; doi:10.1038/s42003-025-07923-1)
Supplement: Supplementary file 2 — Reporting Summary [file 42003_2025_7923_MOESM2_ESM.pdf]

nature portfolio

Corresponding author(s): Mathieu Lesourd

Last updated by author(s): Dec 3, 2024

Reporting Summary

Nature Portfolio wishes to improve the reproducibility of the work that we publish. This form provides structure for consistency and transparency in reporting. For further information on Nature Portfolio policies, see our Editorial Policies and the Editorial Policy Checklist.

Statistics

For all statistical analyses, confirm that the following items are present in the figure legend, table legend, main text, or Methods section.

n/a	Confirmed	
☐	☒	The exact sample size (n) for each experimental group/condition, given as a discrete number and unit of measurement
☐	☒	A statement on whether measurements were taken from distinct samples or whether the same sample was measured repeatedly
☐	☒	The statistical test(s) used AND whether they are one- or two-sided<br>*Only common tests should be described solely by name; describe more complex techniques in the Methods section.*
☐	☒	A description of all covariates tested
☐	☒	A description of any assumptions or corrections, such as tests of normality and adjustment for multiple comparisons
☐	☒	A full description of the statistical parameters including central tendency (e.g. means) or other basic estimates (e.g. regression coefficient) AND variation (e.g. standard deviation) or associated estimates of uncertainty (e.g. confidence intervals)
☐	☒	For null hypothesis testing, the test statistic (e.g. F, t, r) with confidence intervals, effect sizes, degrees of freedom and P value noted<br>*Give P values as exact values whenever suitable.*
☒	☐	For Bayesian analysis, information on the choice of priors and Markov chain Monte Carlo settings
☒	☐	For hierarchical and complex designs, identification of the appropriate level for tests and full reporting of outcomes
☐	☒	Estimates of effect sizes (e.g. Cohen's d, Pearson's r), indicating how they were calculated

Our web collection on statistics for biologists contains articles on many of the points above.

Software and code

Policy information about availability of computer code

Data collection	Python, PsychoPy
Data analysis	Matlab (MathWorks), SPM12, R

For manuscripts utilizing custom algorithms or software that are central to the research but not yet described in published literature, software must be made available to editors and reviewers. We strongly encourage code deposition in a community repository (e.g. GitHub). See the Nature Portfolio guidelines for submitting code & software for further information.

Data

Policy information about availability of data

All manuscripts must include a data availability statement. This statement should provide the following information, where applicable:

- Accession codes, unique identifiers, or web links for publicly available datasets
- A description of any restrictions on data availability
- For clinical datasets or third party data, please ensure that the statement adheres to our policy

The data that support the findings of this study are openly available in OSF at https://osf.io/yh7tm/. Stimulus materials and code are available upon reasonable request.

Research involving human participants, their data, or biological material

Policy information about studies with human participants or human data. See also policy information about sex, gender (identity/presentation), and sexual orientation and race, ethnicity and racism.

Reporting on sex and gender	All data are reported in terms of Sex (female/Male) and the samples of the 3 experiments are balanced according this variable.<br>As we did not have any hypotheses on sex-related effects and as no results in the litterature are reported according to sex-related effects in action tool knowledge tasks, we did not include these analyses in the present work.
Reporting on race, ethnicity, or other socially relevant groupings	No race, ethnicity, or other socially relevant groupings were made in our study.
Population characteristics	See "Resarch sample" section
Recruitment	Experiment 3 (fMRI): participants were recruited from Lyon 2 university (France)<br>Experiment 1: patients were recruited from the neurovascular unit (Besançon) following their stroke. They were tested by neuropsychologists blind to the hypotheses.<br>Experiment 2: Participants were recruited from University of Franche-Comté (France)
Ethics oversight	All experiments were in line with the Declaration of Helsinki.<br>The Experiment 3 was approved by the French Ethics Committee (N°ID-RCB: 2018-A00734-51).<br>The Experiment 1 was approved by the local ethics authorities of Bourgogne Franche-Comté University (CERUBFC-2022-02-15-006)

Note that full information on the approval of the study protocol must also be provided in the manuscript.

Field-specific reporting

Please select the one below that is the best fit for your research. If you are not sure, read the appropriate sections before making your selection.

☐ Life sciences ☒ Behavioural & social sciences ☐ Ecological, evolutionary & environmental sciences

For a reference copy of the document with all sections, see nature.com/documents/nr-reporting-summary-flat.pdf

Behavioural & social sciences study design

All studies must disclose on these points even when the disclosure is negative.

Study description	The study contains 3 quantitative experiments. In experiment 3, fMRI contrasts are used. In experiment 1, ANOVA and single case methodology are carried out on patients' data. In experiment 2, multiple regressions are carried out on responses times in healthy participants.
Research sample	Experiment 3: 20 females, 24.2 years old, 34 right-handers<br>Experiment 1: 30 Left brain-damaged patients (age = 62.4 years old, 15 females, 27 right handers, lesion volume = 9.38 cm3, interval lesion onset = 127.6 days)<br>30 right brain-damaged patients (age = 62.53 years old, 16 females, 30 right handers, lesion volume = 22.85 cm3, interval lesion onset = 133 days)<br>30 controls (age = 59.17 years old, 19 females, 28 right handers)<br>Experiment 2: 52 participants (age = 20.19 years old, 37 women, 52 right handers)
Sampling strategy	The conditions explored in the present study did not have been investigated in previous works (see Lesourd et al., 2021 for a review). As we expected that effect size would be small between our two conditions, we choose to increase the number of subjects compare to similar studies in the litterature on object use. In experiment 2, we conducted multiple regression analyses and there is no simple way to compute sample size, thus we choose to include about 50 participants.<br>Except for experiment 1, where brain-adamged patients were recruited at the hospital, healthy participants were recruited randomly in University of Franche-Comté (Experiment 2) and in Lyon 2 University in France (Experiment 3).
Data collection	Experiment 3: data were collected inside fMRI scanner and PsychoPy was used for behavioral data<br>Experiment 1: the tasks were presented on a laptop and the response from the patients were collected on an Excel file<br>Experiment 2: PsychoPy was used for task presentation and data collection
Timing	Experiment 3: December 2018 - January 2019<br>Experiment 1: March 2022 - February 2023<br>Experiment 2: January 2022 - March 2022
Data exclusions	In experiment 2, multiple regressions were conducted, and outliers (i.e., influential observations) were identified with Cook's distance and removed from the analysis.

Non-participation | In the experiment 1, two patients choose to cancel their participation in the study.

Randomization | Except for the variable Group of the experiment 1 (Left brain vs Right brain damaged patients), all variables were within-subject variables. Thus, there was no randomization.

Reporting for specific materials, systems and methods

We require information from authors about some types of materials, experimental systems and methods used in many studies. Here, indicate whether each material, system or method listed is relevant to your study. If you are not sure if a list item applies to your research, read the appropriate section before selecting a response.

Materials & experimental systems

n/a	Involved in the study
☒	☐ Antibodies
☒	☐ Eukaryotic cell lines
☒	☐ Palaeontology and archaeology
☒	☐ Animals and other organisms
☒	☐ Clinical data
☒	☐ Dual use research of concern
☒	☐ Plants

Methods

n/a	Involved in the study
☒	☐ ChIP-seq
☒	☐ Flow cytometry
☐	☒ MRI-based neuroimaging

Plants

Seed stocks | *Report on the source of all seed stocks or other plant material used. If applicable, state the seed stock centre and catalogue number. If plant specimens were collected from the field, describe the collection location, date and sampling procedures.*

Novel plant genotypes | *Describe the methods by which all novel plant genotypes were produced. This includes those generated by transgenic approaches, gene editing, chemical/radiation-based mutagenesis and hybridization. For transgenic lines, describe the transformation method, the number of independent lines analyzed and the generation upon which experiments were performed. For gene-edited lines, describe the editor used, the endogenous sequence targeted for editing, the targeting guide RNA sequence (if applicable) and how the editor was applied.*

Authentication | *Describe any authentication procedures for each seed stock used or novel genotype generated. Describe any experiments used to assess the effect of a mutation and, where applicable, how potential secondary effects (e.g. second site T-DNA insertions, mosiacism, off-target gene editing) were examined.*

Magnetic resonance imaging

Experimental design

Design type | Block design

Design specifications | For the Tool-Tool compatibility task, a set of 27 black colored line-drawings of uni-manually manipulable tools (targets) were used. Participants saw the picture of the target tool presented with either a related item or an unrelated item. We created a set of 27 unrelated items and 27 related items. Thus, there was a total of 54 pairs of items, half related pairs and half unrelated pairs. In each stimulus, the two items were surrounded by a black frame and appeared on either side of a black cross presented on the center of the screen. Participants were asked to imagine if the two tools are manipulated in the same way. A control condition including 16 new pairs of unrelated items (Control_Tool-Tool), was also constructed. No pairs used in the control condition were previously seen in the experimental condition. Each item pair of the control condition has been prepared in such a way that half of the pair of items included at least one item with a significant colored black surface (e.g., gun grip), which was not the case on the other half pair of items. Participants were asked to explore the items to find the presence or not of a black colored surface on one of the two items. A blocked within-subject design was used with alternating systematically experimental blocks (duration = 24s, 28s or 32s) and baseline periods (19s). During each baseline period, participants were shown a white screen with a fixation cross. Items were blocked by condition (i.e., Tool-Tool and Control_Tool-Tool), with trials varying from 7 to 9 per block. Each stimulus duration was fixed and lasted for 4s. There was no inter-stimulus interval. Six blocks were presented for Tool-Tool condition and four blocks for the control condition, yielding a total of 10 blocks. Although each block varied from 24, to 28, to 32 seconds, after completing all 10 blocks, the average block length was similar for each condition (28 seconds). The number of related and unrelated pairs of stimuli was the same for each experimental condition and was balanced across all the blocks of the experiment . There was the same number of items per condition. The order of the stimuli was randomized, while the order of blocks was pseudo-randomized to ensure that the experimental blocks were homogeneously distributed across the run. Participants were asked to answer only on the last trial of a block, indicated by a red frame surrounding the whole item. Participants indicated their response by pressing one of two buttons with the thumb of their right hand and indicated a 'yes' response with the left button and a 'no' response with the right button.
For the Hand-Tool compatibility task, a set of 24 items was created, each item consisting of a picture of a familiar tool

(e.g., scissors, stapler, nutcracker, etc.) and two pictures of a right hand, each of them depicting a different gesture among four prototypical gestures (precision grip, power grip, trigger, squeeze). Each tool was different whereas the images of the hands were drawn from the same set of four prototypical gestures throughout the experiment. Participants had to choose the image depicting the appropriate hand posture for using the tool. The control condition for the Hand-Tool task (Control_Hand-Tool) was composed of 2 sets of 24 pairs of items: either two tools or two hand postures. The two items could be the same or different. They were drawn from the Hand-Tool condition, i.e. the 24 tools and the 4 prototypical gestures. In each set of pairs, the proportion of identical items was set to 50%. For the experimental and the control conditions, blocks could be 35.4-seconds (2 blocks), 40.1-seconds (2 blocks), or 44.8-seconds (2 blocks) long. Each block consisted of 7, 8, or 9 trials, and each trial in the Hand-Tool condition started by the presentation of the image of a familiar tool for 2 seconds, then a white screen separated items for 0.2 seconds, then the image of a hand posture for the next 2 seconds, followed by a black screen separating trials for 0.5 seconds. For the last trial of each block, signaled with a red frame around the second picture, 3 additional seconds were given to allow for motor response. For the Control_Hand-Tool condition, the two pictures could be either, with the same probability, two tools or two hand postures. In both conditions, trials had a 50% chance of being congruent trials (the tool can be used with this hand posture / the two images are the same). For all conditions, participants were instructed to perform mentally the task but to answer physically only for the last trial of each block, indicated by a red frame surrounding the whole item. The answer had to be given by pushing with the thumb on one of the two buttons of an fMRI-compatible button box.

Behavioral performance measures: correct button press was collected to the participants at the end of each block when the trial was surrounded by a red frame. The correctness of the answers were not analysed.

Acquisition

Imaging type(s): functional and structural

Field strength: 3T

Sequence & imaging parameters: Imaging data were acquired on a 3T Siemens Prisma Scanner (Siemens, Erlangen, Germany) using a 64-channel head coil. Blood-Oxygen Level Dependent (BOLD) images were recorded with T2*-weighted echo-planar images (EPI) acquired with the multi-band sequence. Functional images were all collected as oblique-axial scans aligned with the anterior commissure–posterior commissure (AC–PC) line with the following parameters: 815 volumes per run, 57 slices, TR/TE = 1400 ms / 30 ms, flip angle = 70°, field of view = 96 x 96 mm2, slice thickness = 2.3 mm, voxel size = 2.3 x 2.3 x 2.3 mm3, multiband factor = 2. Structural T1-weighted images were collected using an MPRAGE sequence (224 sagittal slices, TR/TE = 3000 / 2.93 ms, inversion time = 1100 ms, flip angle = 8°, 224 x 256 mm FOV, slice thickness = 0.8 mm, voxel size = 0.8 x 0.8 x 0.8 mm3)

Area of acquisition: functional: field of view = 96 x 96 mm2
structural: 224 x 256 mm2 FOV

Diffusion MRI ☐ Used ☒ Not used

Preprocessing

Preprocessing software: brain extraction (CAT12)
coregistration, normalization, smoothing(SPM12)

Normalization: 4th degree B-Spline interpolation

Normalization template: MNI152

Noise and artifact removal: 3D motion correction and linear detrending, realignment to the mean EPI image with 6-head motion correction parameters and unwarping using topup

Volume censoring: *Define your software and/or method and criteria for volume censoring, and state the extent of such censoring.*

Statistical modeling & inference

Model type and settings: mass univariate

Effect(s) tested: t-tests were mainly used: Hand-Tool > Control; Tool-Tool vs Control; Hand-Tool vs Tool-Tool; and Tool-Tool > Hand-Tool

Specify type of analysis: ☒ Whole brain ☐ ROI-based ☐ Both

Statistic type for inference (See Eklund et al. 2016): cluster-wise

Correction: FWE correction ($p < .05$)

Models & analysis

n/a	Involved in the study
☒	☐ Functional and/or effective connectivity
☒	☐ Graph analysis
☒	☐ Multivariate modeling or predictive analysis
